# Results of a Clinical Scoring System Regarding Symptoms and Surgical Treatment of Isolated Unilateral Zygomatico-Orbital Fractures: A Single-Centre Retrospective Analysis of 461 Cases

**DOI:** 10.3390/jcm11082187

**Published:** 2022-04-14

**Authors:** Lucas M. Ritschl, Matthias Wittmann, Achim von Bomhard, Steffen Koerdt, Tobias Unterhuber, Victoria Kehl, Herbert Deppe, Klaus-Dietrich Wolff, Thomas Mücke, Andreas M. Fichter

**Affiliations:** 1 Department of Oral and Maxillofacial Surgery, School of Medicine, Technical University of Munich, Klinikum Rechts der Isar, Ismaninger Straße 22, 81675 Munich, Germany; matze.wittmann@gmail.com (M.W.); achim.bomhard@tum.de (A.v.B.); tobias.unterhuber@tum.de (T.U.); herbert.deppe@tum.de (H.D.); klaus-dietrich.wolff@tum.de (K.-D.W.); andreas.fichter@tum.de (A.M.F.); 2 Department of Oral and Maxillofacial Surgery, Berlin Institute of Health, Charité–Universitätsmedizin Berlin, Corporate Member of Freie Universität Berlin, Humboldt-Universität zu Berlin, 13353 Berlin, Germany; steffen.koerdt@charite.de; 3 Institute of Medical Informatics, Statistics and Epidemiology, School of Medicine, Technical University of Munich, Grillparzerstrasse 18, 81675 Munich, Germany; victoria.kehl@tum.de; 4 Department of Oral and Maxillofacial Surgery, St. Josefs Hospital, 47441 Moers, Germany; th.mucke@gmx.de

**Keywords:** zygomatic fracture, zygomatico-orbital fracture, orbital fracture, complications, scoring system

## Abstract

Systematic assessment of computed tomography (CT) scans and clinical symptoms is necessary to quickly indicate the correct treatment of zygomatico-orbital (ZMO) fractures. For this purpose, a clinical scoring system (=Clinical Score) was developed and correlated with CT scans to analyse its validity. Every operated, isolated, and unilateral ZMO fracture between January 2012 and December 2016 was screened retrospectively, including patient and treatment data. All available CT scans were analysed, and the grade of dislocation was measured for each case and plane. Four hundred and sixty-one cases were included and showed a median surgery time of 66.0 min (5.0–361.0) and a median postoperative hospital stay of three days (0–25). The distribution of gender, aetiologies and age groups was significantly different (each *p* = 0.001), and the aetiology had a significant influence on the Clinical Score (*p* = 0.038). The degree of dislocation in the coronary and sagittal planes correlated significantly with the Clinical Score with regard to the orbital involvement (*p* < 0.001, ρ = 0.566; *p* < 0.001, ρ = 0.609). The simple, quick, and easy-to-apply Clinical Score showed a significant correlation with the most important planes in CT scans as well as with the clinical course. It may facilitate fast risk stratification of the patient. However, the validity of the proposed score in determining indications must now be evaluated in a prospective setting, including both operated and non-operated fractures.

## 1. Introduction

Isolated zygomatico-orbital (ZMO) fractures represent the second most frequent fracture pattern of the facial skull after nasal bone fractures [1,2]. This fracture may result in functional and aesthetic problems [3]. The first clinical consequences include, most predominantly, double vision, dysfunction of the infraorbital nerve (V_2_) and reduced mouth opening. Often, patients are first seen by a general or trauma surgeon in the emergency room of a secondary or tertiary hospital without the treating specialty (cranio-maxillofacial, plastic or ear-nose-throat (ENT) surgeon) present. Further, with wider broadband deployment, consultations for immobile and/or elderly patients are more and more often carried out teleradiologically. Based on this development, knowledge of the socio- and demographic population, aetiologies, consecutive symptoms and corresponding radiological presentation is valuable for their treatment. As stated by Ellis and Perez, correct clinical and radiological examination are the cornerstones of the three critical points that must be addressed when ZMO fractures are operated, namely, correct and stable anatomic reduction and adequate internal orbital reconstruction [3].

Accordingly, different scoring systems have attempted to summarise the complexity of facial fractures and also isolate ZMO fracture dislocation and clinical symptoms in different ways and with different weightings. The AO CMF (“Arbeitsgemeinschaft für Osteosynthesefragen” Foundation, subdivision CranioMaxilloFacial) recently published a highly sophisticated rating system for midfacial fractures—including the lower central midface with ZMO fractures—based on a hierarchical three-level classification with increasing levels of complexity [4,5]. However, while this sophisticated classification is perfectly suited as a reference for forensic studies, its complexity and lack of clinical aspects can be problematic in the clinical decision-making process. A scoring system that can be applied quickly and reliably even by a person not familiar with the discipline and fracture pattern should not be based on special examinations and signs but on rudimentary symptoms that are easy to evaluate. These findings could accelerate triage in the emergence room, improve communication with a responsible specialist and facilitate the judgement of the temporal urgency of a patient’s supply in the case of external inquiries.

The purpose of this study was to develop a simple clinical scoring system (Clinical Score), which is based on the retrospective analysis of isolated, operated ZMO fractures in a single German centre for Oral and Maxillofacial Surgery and to validate it with the corresponding aetiologies and CT scans.

## 2. Materials and Methods

### 2.1. Ethical Statement and Patient Recruitment

All clinical investigations and procedures were conducted according to the principles expressed in the Declaration of Helsinki. Written patient consent was obtained for surgical treatment as well as any retrospective, anonymous analysis. The retrospective analysis was approved by the Ethical Committee of the Technische Universität München (Approval No. 429/18 S-KK).

Any patient requiring an open reduction and internal fixation (ORIF) of the diagnosed isolated and unilateral ZMO fracture between 1 January 2012 and 31 December 2016 in our department was included in this retrospective study. Patients with additional facial fractures (especially medial orbital wall involvement), infected fractures, malign metastatic disease, neurologic disease, history of previous fractures in this area or those under the age of 18 were excluded. No further exclusion criteria were applied. The records, datasets, and computed tomography (CT) scans of all enrolled patients were screened.

### 2.2. Data Acquisition

The registered data included the following: general information (gender, age, ASA-status (American Society of Anesthesiologist)), aetiology, occurrence of complications and postoperative hospital stay [day]. Available CT scans of the enrolled patients were collected and analysed in a standardised pattern in all three planes with a special focus on the degree of fracture dislocation [mm] at the following typical locations: latero- and infraorbital rim, orbital floor, zygomatico-maxillary buttress and zygomatic arch. For this purpose, no special program or mirroring/symmetrisation from the healthy side was applied. Only the distance from dislocated cortical bone to the adjacent, non-fractured cortical bone was measured.

### 2.3. Scoring System: Clinical Score

A simple scoring system based on clinical findings was developed in which the various criteria received the following different weightings (0–2 points): decrease in visual acuity and double vision (2 points each), dysfunction of V_2_, palpable bone discontinuity and reduced mouth opening (1 point each). This resulted in a range of 0–7 points per case (Table 1).

Other aspects such as flattening of the zygoma area and antimongoloid descent of the external canthus and depression in the area of the zygomatic arch were not rated, even they represented relevant symptoms that help to indicate surgery. However, the idea was to summarise a score based on simple clinical symptoms (Clinical Score) that did not require special knowledge, qualification or specialisation. In the context of trauma, there are various reasons for decreased visual acuity and double vision, which may be swelling- or fracture-related. The former disappears with time and may require clinical ophthalmological control. Fracture-related double vision requires surgical treatment, otherwise the symptom cannot be expected to resolve. This in turn would have severe consequences in daily living, ranging from an increased risk of falling to a ban on driving. For this reason, both symptoms (decrease in visual acuity and double vision) were rated with 2 points each.

### 2.4. Statistical Analysis

The occurrence of complications was described by treatment group using absolute and relative frequencies. Hypothesis testing of differences between subgroups was performed using the Fisher’s exact test. Bivariate Spearman rank correlation coefficients (ρ) were computed to detect relations between clinical symptoms, the calculated Clinical Score, CT-morphological fracture dislocation, duration of operation, hospital stay and incidence of complications. All statistical tests were performed on an exploratory two-sided 5% significance level. No adjustment for multiple testing was incorporated. Analysis was performed with IBM SPSS 24 for Windows software (IBM Corp, Armonk, NY, USA).

## 3. Results

### 3.1. Enrolled Patients and Aetiology

Four hundred and sixty-one patients met the inclusion criteria and were analysed. The age and overall gender distribution are displayed in Table 2.

**Figure 1:**
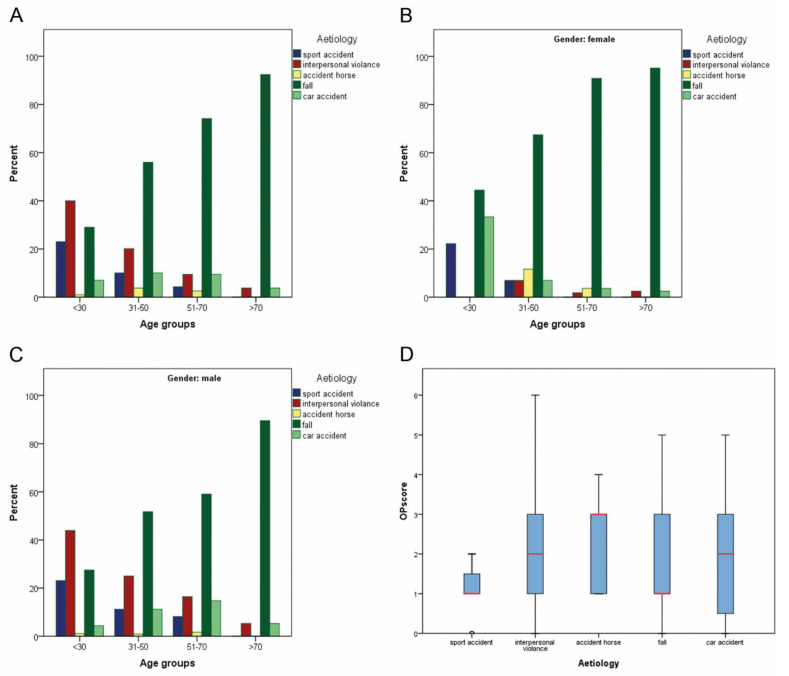
Overview of all aetiologies for surgically treated zygomatico-maxillary complex fractures within four age groups without (**A**) and with consideration of genders (**B**,**C**). Fisher exact testing revealed a significant difference (**A**–**C**, each *p* < 0.001) for the distribution of aetiologies within the age groups. Kruskal–Wallis testing showed a significant influence of the aetiology on the Clinical Score (**D**, *p* = 0.038).

The aetiologies (fall, interpersonal violence, sports-related accident, road traffic accident, horse-related accident and undefined) are shown in Table 3 and Table 4 and Figure 1. The distribution of aetiologies was significantly different in the age groups (*p* = 0.001). A highly significant difference was also detected in the distribution of gender, aetiologies and age groups (each *p* = 0.001). The aetiology also had a significant influence on the Clinical Score (*p* = 0.038, Figure 1D).

### 3.2. Pre- and Postoperative Clinical Symptoms, Clinical Scores

Preoperative clinical findings are listed in Table 5. Dysfunction of the V_2_ was the most common finding (58.1%), followed by double vision (37.5%) and reduced mouth opening (13.0%). Nulla lux was found in 5.0% and other bulbar complications in 3.3% of cases.

**Figure 2:**
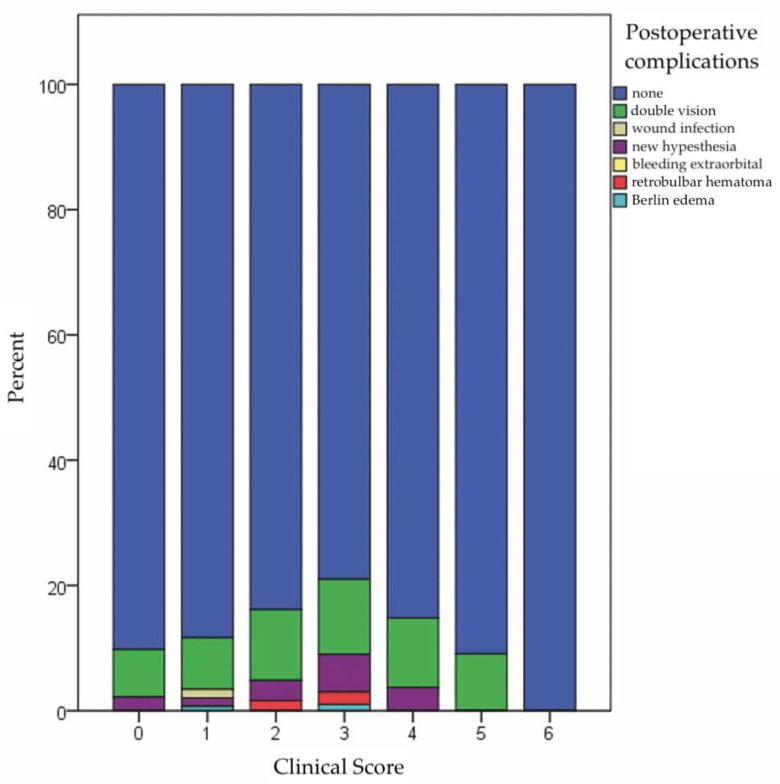
Bar chart depicting the Clinical Score and the associated distribution of recorded postoperative complications (seven points is not displayed because no patient had a Clinical Score of seven points).

The median duration of surgery was 66.0 min (5.0–361.0 min) and two plates were used for sufficient ORIF (0–7 plates). The median duration of postoperative hospital stay was three days (0–25 days). Postoperatively, 43 patients suffered from double vision (9.3%), 13 patients from newly acquired dysfunction of the V_2_ (2.8%), three patients from retrobulbar hematoma (0.7%) and two patients each from wound infection and Berlin edema (each 0.4%) (Figure 2).

The duration of operation and hospital stay correlated significantly with the Clinical Score (*p* < 0.001, ρ = 0.222 and *p* = 0.003, ρ = 0.138). The incidence of postoperative complications did not correlate significantly with the Clinical Score (*p* = 0.053, ρ = 0.092), whereas a significant correlation was found with the duration of operation and hospital stay (*p* = 0.025, ρ = 0.106 and *p* = 0.009, ρ = 0.124) (Table 6).

### 3.3. Degree of Bone Displacement in CT Scan and Correlation Analyses

The distribution of ZMO fractures associated with bone displacement with a special regard to age group and gender is displayed in Table 7. Sinus wall displacement was significantly higher in males <30 and between 51 and 70 years of age in the sagittal view (*p*-values: 0.001 and 0.02, respectively). Other locations and views showed no significant difference between genders within the age groups.

Table 8 displays the results of the bivariate Spearman rank correlation coefficients (ρ). The coronary and sagittal planes correlated significantly with the calculated Clinical Score with regard to orbital involvement (*p* < 0.001, ρ =0.566; *p* < 0.001, ρ = 0.609). The Clinical Score also correlated highly significantly with the displacement of the zygomatico-maxillary buttress in the coronary plane (*p* < 0.001, ρ = 0.178). These three planes were therefore the most relevant for preoperative double vision (*p* < 0.001, ρ = 0.652; *p* < 0.001, ρ = 0.689; *p* < 0.02, ρ = 0.110, respectively), nulla lux (*p* = 0.007, ρ = 0.128; *p* = 0.013, ρ = 0.118; *p* = 0.917, ρ = −0.005, respectively) and preoperative dysfunction of the V_2_ (*p* = 0.006, ρ = 0.130; *p* < 0.001, ρ = 0.185; *p* < 0.001, ρ = 0.306, respectively). Reduced mouth opening, on the other hand, highly significantly correlated with the degree of dislocation in the axial plane (*p* <0.001, ρ = 0.179).

## 4. Discussion

### 4.1. Aetiology

The aetiology of ZMO fractures shows locoregional differences. In contrast to an Italian or Brazilian patient cohort [6,7], where motor vehicle accidents were the most common reason for a ZMO fracture, in our Bavarian cohort, aetiologies followed a typical age distribution as follows: While the proportion of interpersonal violence and sporting accidents decreased, the incidence of falls increased with age (Table 3, Figure 1A–C). In line with previous research [8,9,10,11], a typical gender distribution with a more frequent male representation in interpersonal violence and sports-related accidents was also seen in our study (Table 4). Among the youngest male age group (<30 years), interpersonal violence was the most common cause. The shift from road traffic accidents to violence has already been pointed out in the literature [12,13]. The exact causes are unclear, but what is known is that vehicle travel has become much safer over time because of airbags, seat belt use, improved vehicle design and stricter drink and drug driving laws. At the same time, an increase in aggressiveness among younger people has been reported.

Interestingly, aetiology had a significant influence on the evaluated Clinical Score (Figure 1D, *p* = 0.038) as follows: Sports accidents and fall events had a mean Clinical Score of one, while traffic accidents and interpersonal violence had a mean score of two and horse accidents had a mean score of three. This can be explained by the magnitude of the force involved. The higher the impact (e.g., traffic accident or horse kick), the higher the degree of dislocation, which in turn translates to the patient’s clinical symptoms.

### 4.2. Clinical Symptoms

In concordance with the literature [6,10], the most commonly observed clinical symptoms of operated ZMO fractures in our study were hypoesthesia/paraesthesia (58.1%), double vision (37.5%) and reduced mouth opening (13.0%). While the percentage of patients with hypesthesia (52.2%) was comparable to our collective, reduced mouth opening (47.3%) was more frequently observed in Hwang et al.’s (2009) cohort, while double vision was reported in only 8.3% of patients. In Calderoni et al.’s (2011) cohort, the incidence of both double vision (13.2%) and paraesthesia (12.1%) was relatively low, while reduced mouth opening (14.3%) was comparable with our study collective. The most logical explanation for these discrepancies between studies might be differing trauma mechanisms and degrees of fracture dislocation.

### 4.3. Clinical Score Analysis

In our study, the Clinical Score was calculated on the basis of the most common clinical symptoms at initial presentation, which were the following: decrease in visual acuity and double vision (2 points each), dysfunction of V_2_, palpable bone discontinuity and reduced mouth opening (1 point each). This resulted in a range of 0–7 points per case (Table 1). The Clinical Score correlated significantly with two important aspects that have not been published in this way in the literature before, namely, (1) the bony dislocation in the CT scan and (2) the operation duration and length of hospital stay. Further, orbital floor involvement also correlated significantly with the Clinical Score, which can be helpful for initial diagnosis and assessment in peripheral hospitals if a CT scan is not available at presentation. These results emphasise the importance of clinical examination, which is always available and can, in its basic features, also be performed by any physician without special training at first presentation at the emergency room or outpatient clinic. Consequently, in the sense of a reasonable and effective triage, it is possible to direct the patient with a valid tentative diagnosis through this focused physical examination to save the most limited resource, “time”. In addition, even without having examined the patient him-/her-/itself, a specialist consulted could initiate further diagnostic measures and more validly weigh the urgency of a presentation to the specialist department.

Higher Clinical Scores correlate with a higher degree of dislocation of the fractures. In such cases, a specialist should be consulted promptly, and three-dimensional imaging should be performed. It is also safe to assume that cases with a high Clinical Score are more likely to be considered for surgery than cases with a low score. However, our results do not allow a statement about the exact value at which a more thorough assessment should take place or can safely be omitted. Nor can our results allow a decision for or against surgery on the basis of a specific score yet. Further, prospective validation of the score is necessary for such decisions.

The median duration of surgery was 66.0 min (5.0–361.0 min) and the median postoperative hospital stay was three days (0–25 days). The range of results displays the mixture of complexity of included, operated and isolated unilateral ZMO fractures. Nevertheless, our hospital stay was lower than the results presented by Hwang and Kim, who described an average hospital stay of 8.7 days [10]. Since the distribution of aetiologies is comparable, health economic considerations are likely primarily responsible for the difference in length of stay. Both the duration of operation and hospital stay correlated significantly with the Clinical Score (*p* = 0.025, ρ = 0.106 and *p* = 0.009, ρ = 0.124, respectively) (Table 6). This information could contribute to a better assessment of the clinical course already at initial presentation.

### 4.4. Comparison to Other Scoring/Rating Systems

A number of other studies have analysed scoring systems for trauma in general and maxillofacial trauma in particular. The main purpose of this study, however, was not to evaluate existing, established universal trauma scoring systems such as the New Injury Severity Score (NISS), the Facial Injury Severity Score (FISS), the Facial Fracture Severity Scale (FFSS) or the Maxillofacial Injury Severity Score (MFISS) [14,15,16,17], but to focus on operated ZMO fractures alone and evaluate the significance of basic clinical assessment.

The FFSS described by Catapano et al. (2010) documents the fracture patterns based on 41 major parts and scoring points from 0–3. Another score that rates maxillofacial fractures is the Craniofacial Disruption Score (CDS) [18]. The CDS is a systematic, hierarchical coding system for fractures of the craniofacial region based on 20 major anatomical regions and reflecting the degree of disruption. More recently, the AO CMF has published a hierarchical three-level classification system for midfacial fractures—including the lower central midface with ZMO fractures—which enables a valid classification for study purposes and forensic investigations [4,5]. The CDS and AO CMF coding systems are both very complex and not appropriate for general surgeons in the emergency room during the initial presentation. These systems should therefore be reserved for specialists during secondary consultation or for study purposes. Both scoring systems have in common that they essentially focus on the (CT-) morphological fracture patterns, ignoring both the soft tissue situation and the clinical symptoms that often represent the leading cause of initial presentation. The MFISS, which is based on the Abbreviated Injury Scale (AIS) and its revision (AIS-90), includes soft tissue injuries as well as the following three functional maxillofacial parameters: malocclusion, limited mouth opening and facial deformity [15]. An evaluation of this scoring system on 902 maxillofacial trauma cases revealed a significant correlation with costs and the number of days of hospital stay.

A study by Chen et al. compared four scoring systems on 28 patients and correlated the results with blinded evaluations of 35 experts [14]. The MFISS showed the highest correlation with the expert scores. This again underlines the relevance of the clinical symptoms besides the purely anatomical rating and assessment. Von Hout et al. have divided ZMO fractures into types A, B, and C based purely on CT morphology [19]. A Type A represents the simplest fracture configuration in terms of an incomplete tripod fracture. A Type B fracture represents a tetrapod fracture and thus includes the zygomatic arch fracture. The complicated ZMO fractures are grouped under type C and include the comminuted fractures. C-type fractures were associated with more complex reconstructions necessitating 3D planning and intraoperative imaging and needed more frequent secondary corrections [19].

Our simple, clinical scoring system correlated significantly with the aetiology and the degree of bone dislocation, especially in the coronary CT planes for judgement of the orbital floor and the latero-orbital wall as well as with the sagittal CT plane for judgement of the lateral sinus wall (Table 8). Further, we also showed a significant correlation between our score with the duration of operation and hospital stay (Table 6). In summary, our scoring system could help the general or trauma surgeon in the emergency room of a secondary or tertiary hospital without the treating specialty to rate and categorise the diagnosed ZMO fracture. Further, a treating specialist (cranio-maxillofacial, plastic or ENT surgeon) could also rate the ZMO fracture easily, even teleradiologically, only by querying the clinical symptoms.

### 4.5. Limitations

The retrospective study design and the fact that only operated cases were included are two major limitations of this study that may weaken the validity of the proposed score. In its current form, the score cannot be used to make a decision for or against surgery without prior validation of the score in a prospective setting including both operated and non-operated cases. Nor is it advisable to refrain from CT imaging solely on the basis of a specific score value. Nevertheless, we think that the unequivocal results support the proposed score and serve as an important basis for further prospective investigations. In addition, the chosen clinical symptoms were not sophisticated (such as flattening of the zygoma area, antimongoloid descent of the external canthus and depression in the area of the zygomatic arch). The idea of this presented Clinical Score to categorise only very simple clinical symptoms that do not require any special knowledge or qualification.

In this presented analysis of pure ZMO fracture, no cases with additional medial orbital wall fractures have been included. This exclusion was performed to reduce the bias increase in double vision and the complexity of surgical exploration and repair [20,21].

A comparison with other studies remains difficult because of local demographic and socioeconomic differences. Nevertheless, this retrospective study analysed surgically treated ZMO fractures of legal-aged patients at one German university hospital and provides current demographic results.

## 5. Conclusions

Clinical examination remains the gold standard in determining indications for surgical treatment of zygomatic fractures. Anatomically weighted systems facilitate communication between experts and enable comparable documentation for study purposes. Clinically weighted systems allow a more global judgement of facial trauma, even if the score is more focused on one type of fracture, as seen in our study. The presented Clinical Score facilitates the assessment of clinical symptoms and correlates with aetiology and the degree of fracture dislocation. The orbital floor involvement correlated significantly with the Clinical Score, which can be helpful for initial diagnosis and assessment in peripheral hospitals if a CT scan is not available at presentation. The validity of the proposed score in determining indications must now be evaluated in a prospective setting, including both operated and non-operated fractures.

## Figures and Tables

**Table 1:** Clinical Score.

Symptom	Score
Decrease in visual acuity	2
Double vision	2
Dysfunction V_2_	1
Palpable bone discontinuity	1
Reduced mouth opening	1
Total	0–7

Abbreviation: V_2_ = infraorbital nerve.

**Table 2:** General information of the 461 retrospectively analysed cases of isolated unilateral zygomatico-orbital fractures included in the study.

Parameter	Age Median (Range)	Gender (M/F)
Total	47.0 (18–90)	312/149
Age group/ Gender distribution	18–30 31–50 51–70 >70	92/9 119/43 62/55 39/42

Abbreviations: M/F = male/female.

**Table 3:** Aetiology of 461 retrospectively analysed cases of isolated unilateral zygomatico-orbital fractures.

		Age Groups								
		<30		31–50		51–70		>70		*p*-Value *
		Count	[%]	Count	[%]	Count	[%]	Count	[%]	
Aetiology	Sports-related accident	23	23.0	16	10.1	5	4.3	0	0.0	<0.001
	Interpersonal violence	40	40.0	32	20.1	11	9.5	3	3.8	
	Horse-related accident	1	1.0	6	3.8	3	2.6	0	0.0	
	Fall	29	29.0	89	56.0	86	74.1	73	92.4	
	Road traffic accident	7	7.0	16	10.1	11	9.5	3	3.8	

* Fisher exact testing with an exploratory two-sided 5% significance level. Seven cases were excluded because of undefined aetiology.

**Table 4:** Aetiology of 461 retrospectively analysed cases of isolated unilateral zygomatico-orbital fractures according to gender and age groups.

				Age Groups								
				<30		31–50		51–70		>70		*p*-Value *
				Count	[%]	Count	[%]	Count	[%]	Count	[%]	
Gender	Female	Aetiology	Sports-related accident	2	22.2	3	7.0	0	0.0	0	0.0	<0.001
			Interpersonal violence	0	0.0	3	7.0	1	1.8	1	2.4	
			Horse-related accident	0	0.0	5	11.6	2	3.6	0	0.0	
			Fall	4	44.4	29	67.4	50	90.9	39	95.1	
			Road traffic accident	3	33.3	3	7.0	2	3.6	1	2.4	
	Male	Aetiology	Sports-related accident	21	23.1	13	11.2	5	8.2	0	0.0	<0.001
			Interpersonal violence	40	44.0	29	25.0	10	16.4	2	5.3	
			Horse-related accident	1	1.1	1	0.9	1	1.6	0	0.0	
			Fall	25	27.5	60	51.7	36	59.0	34	89.5	
			Road traffic accident	4	4.4	13	11.2	9	14.8	2	5.3	

* Fisher exact testing with an exploratory two-sided 5% significance level. Seven cases were excluded because of undefined aetiology.

**Table 5:** Preoperative clinical findings in 446 cases of isolated unilateral zygomatico-orbital fractures.

Preoperative Symptoms	Yes/No/Unknown (Yes %)
Reduced eye motility	41/405/0 (8.9%)
Double vision	173/273/0 (37.5%)
Anisocoria	8/438/0 (1.7%)
Dysfunction V_2_	268/178/0 (58.1%)
Emphysema	23/412/9 (5.0%)
Reduced mouth opening	60/377/24 (13.0%)
Bulbar complication	15/420/11 (3.3)
Nulla lux	23/423/0 (5.0%)

Abbreviation: V_2_ = infraorbital nerve.

**Table 6:** Computed bivariate Spearman rank correlation coefficients (ρ) of Clinical Score and therapy-specific course.

			Clinical Score	Operation Duration	Hospital Stay	Postoperative Complications
Spearman’s rho	Clinical Score	Correlation coefficient	1.000	0.222 **	0.138 **	0.092
		Sig. (2-tailed)		0.000	0.003	0.053
	Operation duration	Correlation coefficient	0.222 **	1.000	0.168 **	0.106 *
		Sig. (2-tailed)	0.000		0.000	0.025
	Hospital stay	Correlation coefficient	0.138 **	0.168 **	1.000	0.124 **
		Sig. (2-tailed)	0.003	0.000		0.009
	Postoperative complications	Correlation coefficient	0.092	0.106 *	0.124 **	1.000
		Sig. (2-tailed)	0.053	0.025	0.009	

* Correlation is significant at the 0.05 level (2-tailed); ** Correlation is significant at the 0.01 level (2-tailed).

**Table 7:** Radiological findings and extent of bony dislocation [mm] and incidence of peribulbar herniation [mm] in isolated unilateral zygomatico-orbital fractures.

Localization	Age Groups	Axial Plane Median (Range)			Coronary Plane Median (Range)			Sagittal Plane Median (Range)		
	[years]	Male	Female	*p*-Value	Male	Female	*p*-Value	Male	Female	*p*-Value
Latero-orbital	<30 31–50 51–70 >70	1.0 (0.0–4.0) 1.0 (0.0–4.0) 1.0 (0.0–6.0) 1.0 (0.0–4.0)	0.0 (0.0–2.0) 1.0 (0.0–3.0) 1.0 (0.0–4.0) 1.0 (0.0–4.0)	0.51 0.08 0.09 0.19	0.3 (0.0–4.0) 1.2 (0.0–4.0) 1.2 (0.0–5.0) 1.5 (0.0–3.0)	0.0 (0.0–2.0) 1.3 (0.0–5.0) 0.0 (0.0–4.0) 1.1 (0.0–6.0)	0.41 0.89 0.39 0.19	not rated in sagittal view	not rated in sagittal view	
Zygomatic arch	<30 31–50 51–70 >70	0.0 (0.0–3.0) 1.0 (0.0–4.0) 1.0 (0.0–4.0) 0.0 (0.0–4.0)	0.0 (0.0–6.0) 0.0 (0.0–3.0) 1.0 (0.0–4.0) 0.0 (0.0–4.0)	0.79 0.24 0.80 0.62	0.0 (0.0–3.0) 1.0 (0.0–3.0) 1.0 (0.0–4.0) 0.0 (0.0–3.0)	0.0 (0.0–4.0) 0.0 (0.0–2.0) 1.0 (0.0–4.0) 0.0 (0.0–3.0)	0.83 0.15 0.64 0.86	0.0 (0.0–2.0) 0.0 (0.0–3.0) 0.0 (0.0–3.0) 0.0 (0.0–2.0)	0.0 (0.0–4.0) 0.0 (0.0–2.0) 0.0 (0.0–1.0) 0.0 (0.0–2.0)	0.85 0.63 0.84 0.21
Zygomatico-maxillary buttress	<30 31–50 51–70 >70	rotation	rotation	/	rotation	rotation		not rated in sagittal view	not rated in sagittal view	
Sinus wall	<30 31–50 51–70 >70	2.5 (0.0–8.0) 2.0 (0.0–10.0) 2.0 (0.0–12.0) 2.0 (0.0–8.0)	0.0 (0.0–2.0) 1.0 (0.0–5.0) 2.0 (0.0–5.0) 2.0 (0.0–5.0)	0.003 0.16 0.02 0.39	2.0 (0.0–7.0) 2.0 (0.0–12.0) 2.0 (0.0–8.0) 2.0 (0.0–9.0)	0.0 (0.0–2.0) 3.0 (0.0–6.0) 2.0 (0.0–5.0) 2.0 (0.0–5.0)	0.001 0.09 0.07 0.35	2.0 (0.0–9.0) 2.0 (0.0–6.0) 2.0 (0.0–10) 2.0 (0.0–6.0)	2.0 (0.0–6.0) 2.0 (0.0–7.0) 1.0 (0.0–6.0) 2.0 (0.0–5.0)	0.73 0.81 0.85 0.48
Orbital floor	<30 31–50 51–70 >70	not rated in axial view	not rated in axial view	/	2.5 (0.0–6.5) 2.5 (0.0–8.8) 2.2 (0.0–7.2) 2.9 (0.0–7.1)	2.1 (0.0–3.2) 2.2 (0.0–6.7) 2.7 (0.0–6.1) 3.1 (0.0–13.6)	0.23 0.46 0.60 0.24	2.5 (0.0–6.7) 2.4 (0.0–8.4) 2.5 (0.0–10.9) 2.4 (0.0–7.6)	2.2 (0.0–4.0) 2.3 (0.0–6.9) 2.5 (0.0–7.0) 3.2 (0.0–9.1)	0.23 0.77 0.80 0.07

Given *p*-values for male vs. female are based on the Mann–Whitney U test with an exploratory two-sided 5% significance level.

**Table 8:** Computed bivariate Spearman rank correlation coefficients (ρ) of Clinical Score and preoperative symptoms with degree of fracture dislocation in corresponding CT scans in patients with isolated unilateral zygomatico-orbital fractures.

			CT Axial Plane Latero-Orbital	CT Axial Plane Zygomatic Arch	CT Coronary Plane Orbital Floor	CT Sagittal Plane Orbital Floor	CT Coronary Plane Lateral Sinus Wall
Spearman’s rho	Clinical Score	Correlation coefficient	0.087	−0.019	0.566 **	0.609 **	0.178 **
		Sig. (2-tailed)	0.067	0.690	0.000	0.000	0.000
	Preoperative double vision	Correlation coefficient	0.050	−0.050	0.652 **	0.689 **	0.110 *
		Sig. (2-tailed)	0.295	0.295	0.000	0.000	0.020
	Preoperative nulla lux	Correlation coefficient	0.018	−0.036	0.128 **	0.118 *	−0.005
		Sig. (2-tailed)	0.704	0.443	0.007	0.013	0.917
	Preoperative reduced eye motility	Correlation coefficient	−0.009	0.078	0.120*	0.090	0.037
		Sig. (2-tailed)	0.846	0.100	0.011	0.058	0.437
	Preoperative anisocoria	Correlation coefficient	0.061	0.027	0.028	0.003	0.037
		Sig. (2-tailed)	0.199	0.574	0.551	0.943	0.435
	Preoperative reduced mouth opening	Correlation coefficient	−0.018	0.179 **	−0.056	−0.055	−0.096 *
		Sig. (2-tailed)	0.701	0.000	0.239	0.251	0.044
	Preoperative hypesthesia V_2_	Correlation coefficient	0.138 **	−0.032	0.130 **	0.185 **	0.306 **
		Sig. (2-tailed)	0.004	0.503	0.006	0.000	0.000
	Preoperative emphysema	Correlation coefficient	−0.049	−0.078	0.050	0.041	0.025
		Sig. (2-tailed)	0.304	0.102	0.295	0.395	0.608
	Preoperative complications	Correlation coefficient	0.109 *	−0.020	0.107*	0.098 *	−0.024
		Sig. (2-tailed)	0.023	0.680	0.026	0.040	0.612

Abbreviation: V_2_ = infraorbital nerve; * Correlation is significant at the 0.05 level (2-tailed); ** Correlation is significant at the 0.01 level (2-tailed).

## Data Availability

The data presented in this study are available on request from the corresponding author.
